# Emerging role of LRRK2 in human neural progenitor cell cycle progression, survival and differentiation

**DOI:** 10.1186/1750-1326-4-25

**Published:** 2009-06-15

**Authors:** Javorina Milosevic, Sigrid C Schwarz, Vera Ogunlade, Anne K Meyer, Alexander Storch, Johannes Schwarz

**Affiliations:** 1 Translational Centre for Regenerative Medicine – Leipzig (TRM-Leipzig), University of Leipzig, Philipp-Rosenthal-Straße 55, 04103 Leipzig, Germany; 2 Department of Neurology, University of Leipzig, Liebigstr. 22a, 04103, Germany; 3 Department of Neuropathology, University of Leipzig, 04103 Leipzig, Germany; 4 Department of Neurology, Dresden University of Technology, 01307 Dresden, Germany; 5 Center for Regenerative Therapies Dresden, Dresden University of Technology, 01307 Dresden, Germany

## Abstract

Despite a comprehensive mapping of the Parkinson's disease (PD)-related mRNA and protein leucine-rich repeat kinase 2 (LRRK2) in the mammalian brain, its physiological function in healthy individuals remains enigmatic. Based on its structural features and kinase properties, LRRK2 may interact with other proteins involved in signalling pathways. Here, we show a widespread LRRK2 mRNA and/or protein expression in expanded or differentiated human mesencephalic neural progenitor cells (hmNPCs) and in post-mortem *substantia nigra *PD patients. Using small interfering RNA duplexes targeting LRRK2 in hmNPCs following their differentiation into glia and neurons, we observed a reduced number of dopaminergic neurons due to apoptosis in LRRK2 knockdown samples. LRRK2-deficient hmNPCs exhibited elevated cell cycle- and cell death-related markers. In conclusion, a reduction of LRRK2 expression in hmNPCs severely impaired dopaminergic differentiation and/or survival of dopaminergic neurons most likely via preserving or reactivating the cell cycle.

## Background

The pathology of Parkinson's disease (PD) involves the loss of dopaminergic neurons (DNs) in the *substantia nigra *and the presence of intraneuronal accumulations of aggregated proteins (Lewy bodies) in surviving neurons. Most PD cases appear to be sporadic. However, several mutations linked to inherited forms of PD have also been identified such as parkin, α-synuclein, PINK, DJ-1 [1]. Mutations in the leucine-rich repeat kinase 2 (*LRRK2, PARK8*) gene are the most common cause of both autosomal-dominant familial and sporadic late-onset cases of PD identified so far [2].

*LRRK2 *encodes for dardarin, a large and complex protein with an approximate molecular weight of 286 kDa that comprises multiple domains, including leucine-rich repeat (LRR), ROC-COR GTPase, mitogen-activated protein kinase kinase kinase (MAPKKK) and WD40 domains [3,4]. These functional domains support a role in cellular signalling. The most common mutation, G2019S within the MAPKKK domain enhances kinase activity, possibly contributing to LRRK2-mediated pathology through a toxic gain-of-function mechanism [5]. This gain of function may contribute to the death of brain cells that produce dopamine [6,7]. LRRK2 has become a prime therapeutic target in respect to neuroprotection or symptomatic treatment of PD. Once the pathogenic mechanism of mutated LRRK2 has been identified, we can search for molecules that are able to repair the signalling defect in dopamine neurons and prevent them from dying.

LRRK2 is a ubiquitous protein, which is constitutively expressed in various brain regions and various cell types including neurons and glia in the human brain [8,9]. Further, it is tightly connected with pathological inclusions in several neurodegenerative disorders [10]. LRRK2 expression is high in dopamine-innervated brain areas [8,11]. While LRRK2 mRNA and protein expression were comprehensively mapped, the normal physiological role of LRRK2 protein and its physiological substrates have not yet been identified.

In this study, we investigated the distribution of LRRK2 in neural progenitor cells (NPCs) during proliferation and differentiation. Using RNA interference technology, we explored the physiological role of LRRK2 protein in NPCs in respect to cell cycle, dopaminergic differentiation and survival.

## Results

### LRRK2 expression in neural progenitors and PD substantia nigra

**Figure 1:**
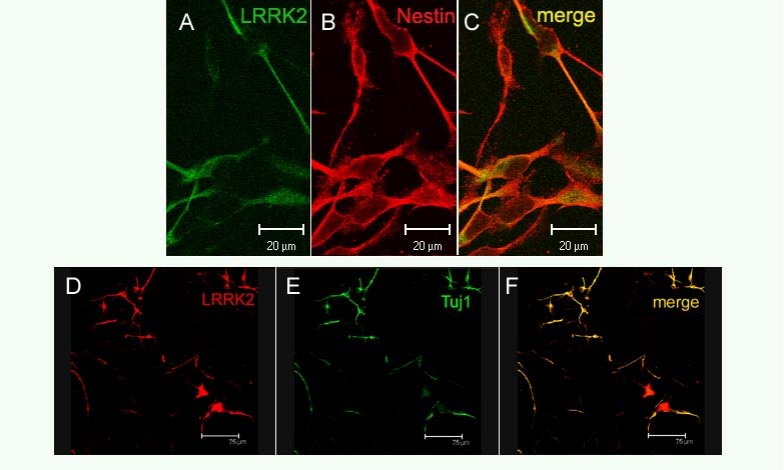
**LRRK2 constitutive expression in neural progenitors and terminally differentiated hmNPCs**. (A-C) LRRK2 (green) is co-expressed with nestin (red) in proliferating hmNPC. (D-F) Upon hmNPCs differentiation, LRRK2 was observed in dopaminergic neurons (TH-positive). Note the cytoplasmic pattern as previously described. Scale bar: 20 μm (A-C), 75 μm (D-F).

**Figure 2:**
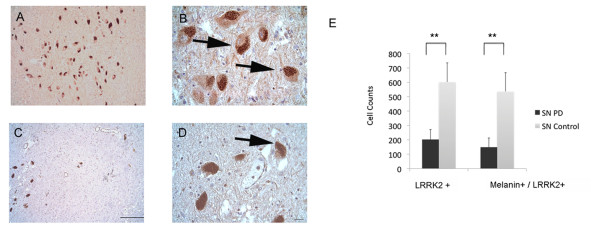
**Microphotograph of *substantia nigra *from controls or PD patients stained for LRRK2**. (A, B) control coronal midbrain paraffin section (5 μm) and (C, D) PD midbrain section in 200× (scale bar 50 μm) or 800× (scale bar 20 μm) magnification displaying the substantial loss of cells expressing melanin and LRRK2. The arrows exemplarily show cells expressing both LRRK2 and melanin. (E) Cell counts of melanin and LRRK2 expressing cells in *substantia nigra *of controls and patients with Parkinson's disease. Note the significant loss of cells expressing melanin and LRRK2 (** *P *< 0.01, unpaired Student's t-test).

In order to explore LRRK2 protein expression in human midbrain-derived NPCs (hmNPCs), we selected an antibody according to a comprehensive characterization of various LRRK2 antibodies [12]. LRRK2 protein expression was examined in several hmNPC preparations that were cultured for various time periods (passage 4–12). 80–95% of these proliferating cells that expressed the NPC marker nestin were also immunoreactive for LRRK2 protein (Fig. 1A–C). Following differentiation, LRRK2 protein was expressed in both, glial cells (data not shown) and neurons (Fig. 1D–F). In addition, LRRK2 was analyzed in midbrain sections of 4 patients with a *post mortem *diagnosis of idiopathic PD (Fig. 2C, D) and 4 controls (Fig. 2A, B). Melanin-positive cells, LRRK2-immunoreactive cells and cells expressing both melanin and LRRK2 were counted within the *substantia nigra*. LRRK2-positive cells were markedly and significantly reduced (-66%; 202.5 ± 69.0 cells) in PD patients compared to controls (601.8 ± 133.2 cells; Fig. 2E). Accordingly, cells expressing both melanin and LRRK2 were significantly reduced in patients (-73%; 148.8 ± 64.1 cells) compared to controls (536.3 ± 130.5 cells; Fig. 2E).

### LRRK2 down-regulation leads to a reduction of TH expression

**Figure 3:**
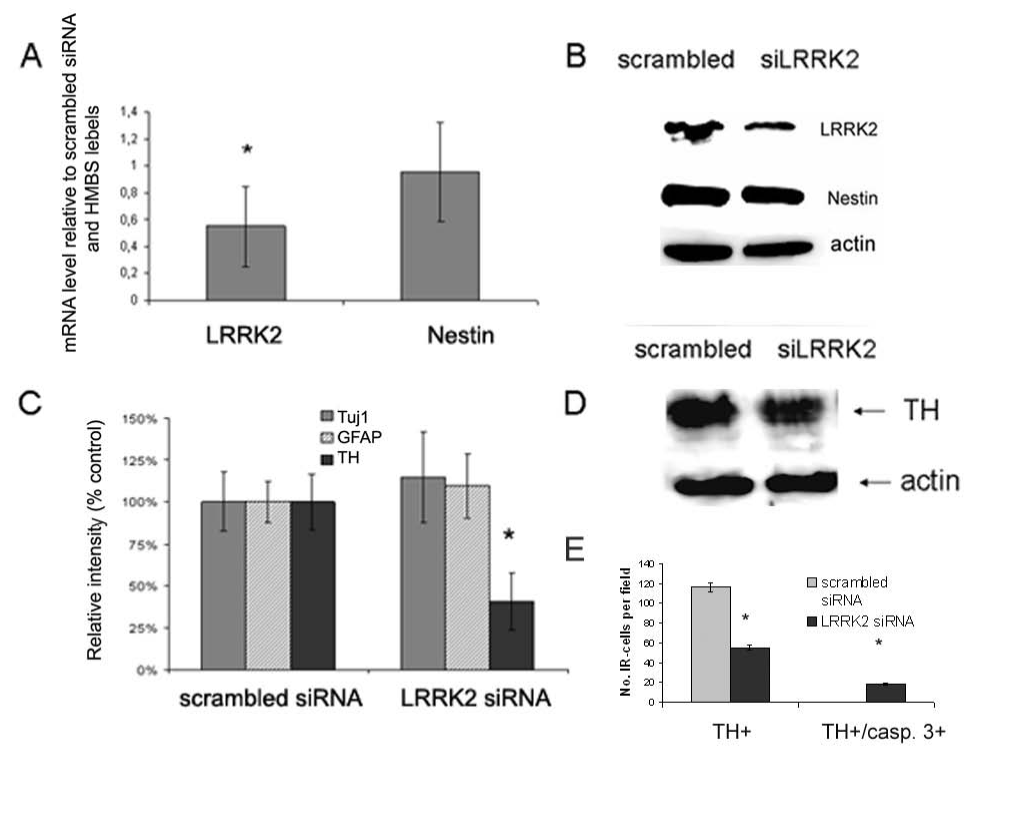
**LRRK2 involvement in dopaminergic differentiation of hmNPCs**. (A) Representative samples of expanded hmNPCs quantified for LRRK2 or nestin mRNA level as determined 72 h upon nucleofection with either non-specific siRNA (scrambled control siRNA) or LRRK2-specific siRNA, both relative to HMBS mRNA expression. (B) Immunoblot demonstrating knockdown of LRRK2 protein expression in hmNPCs, observed 72 h after siRNA delivery. (C) Densitometric analysis of Western blots exhibiting relative band intensities of unaffected neuronal (Tuj1) and glial (GFAP) markers but reduced TH expression, all normalized to control samples. β-actin served as loading control (D). Quantification of TH+ and TH/caspase-3 double-positive cells expressed as number of immunoreactive cells per visual field (n = 6). Data are means of 3 independent experiments ± S.E.M. *P < 0.05 vs. scrambled siRNA sample.

Semi-quantitative RT-PCR analysis (relative to HMBS expression as a house keeping gene) of siLRRK2 vs. scrambled (non-silencing control) siRNA samples revealed a reduction in LRRK2 mRNA by 54.9 ± 30.1% following nucleofection and 72 h expansion using standard conditions. By contrast, the mRNA level of the NPC marker nestin did not exhibit a significant difference compared to scrambled control samples (95.3 ± 37%). RT-PCR data were confirmed using immunoblotting and densiometric quantification of bands demonstrating a significant LRRK2 protein knockdown (43 ± 11% of control; *P *= 0.003, t-test) and a non-significant nestin down-regulation (80 ± 1%; Fig. 3A) in expanding hmNPCs, 72 h post-nucleofection. The knockdown of LRRK2 in hmNPCs following 2 weeks of differentiation did not significantly affect neuronal (Tuj1) or glial (GFAP) differentiation, but resulted in a reduced capacity to differentiate into dopaminergic neurons (40 ± 17% of control scrambled siRNA; Fig. 3C, D). The number of TH-immunoreactive (TH-IR) cells per field in LRRK2 siRNA transfected was reduced to 55.0 ± 2.6 vs. 116.7 ± 4.6 cells counted in scrambled control samples (Fig. 3E).

### LRRK2 deficient dopaminergic neurons die presumably by apoptosis

**Figure 4:**
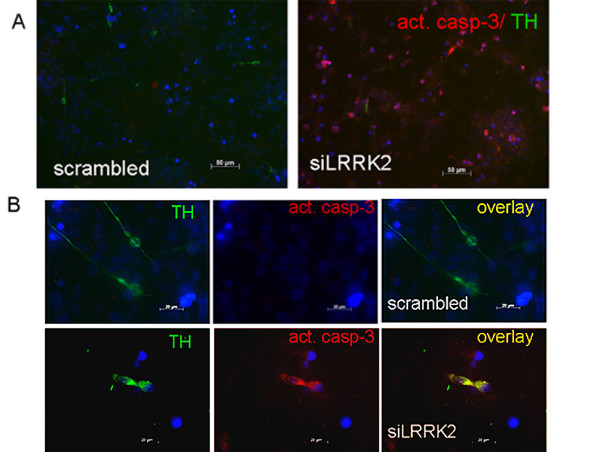
**LRRK2 down-regulation in differentiated neuralprogenitors induces cell death**. (A) Partial LRRK2 protein reduction induced appearance of dying cells as detected by immunostaining against cleaved caspase-3. Note plenty of red cells expressing activated caspase-3 at lower magnification (A – right panel). Caspase-3 containing cells were not observed in hmNPCs treated with scrambled siRNA before differentiation (A – left panel). (B) Some of the TH^+ ^cells in siLRRK2 cultures were also positive for acivated caspase-3 (higher magnification).

To assess the potential loss of dopaminergic neurons by apoptosis scrambled and siRNA treated hmNPCs cultures were stained for cleaved caspase-3 and TH (double immunocytochemistry). Caspase-3 is considered to execute apoptotic cell death in many cell types including dopaminergic neurons when activated via cleavage [13,14]. Cells transfected with scrambled control RNA did not show activated caspase-3 expression. By contrast, siLRRK2 expressing differentiated hmNPCs exhibited overt cell death (Figure 4A). On closer inspection, cleaved caspase-3-positive cells revealed TH/caspase-3 co-expression indicating a loss of dopaminergic cells via apoptosis (Fig. 4B). Counting of TH/caspase-3 double-positive cells has revealed 17.7 ± 0.9 TH+ cells with activated caspase-3 per visual field (Fig. 3E).

### Cell cycle preservation or reactivation in LRRK2 deficient hmNPCS

**Figure 5:**
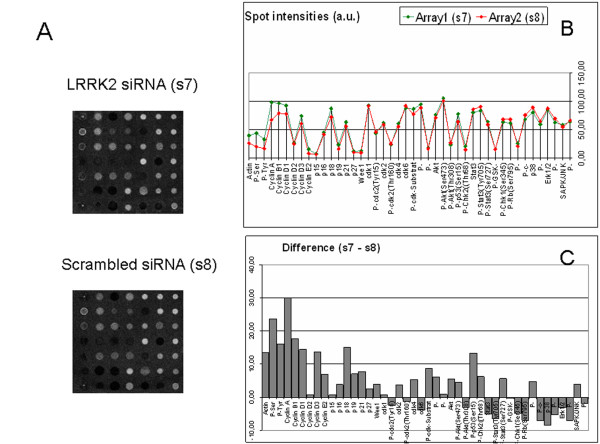
**Cell cycle reactivation and cell death induction in LRRK2 deficient hmNPCs**. Human mesencephalic neuroprogenitors were nucleofected with corresponding siRNAs (scrambled or LRRK2 siRNA) and differentiated for 2 weeks after a 24 h recovery period. Protein extracts were subjected to antibody microarray (*Signal.screen *44 F employing 44 antibodies belonging to signalling cascades: MAPK, Akt/PKB, GSK, cell cycle regulation, apoptosis and transcription factors). Here we illustrate the comparison of sample 7 (LRRK2 siRNA) vs. sample 8 (scrambled siRNA): (A) the original spot intensities, (B) the differences and (C) selected averages presented in arbitrary units (a. u.). The differences in the intensities lower 10 a.u. should be neglected since they lay outside the test reliability.

**Figure 6:**
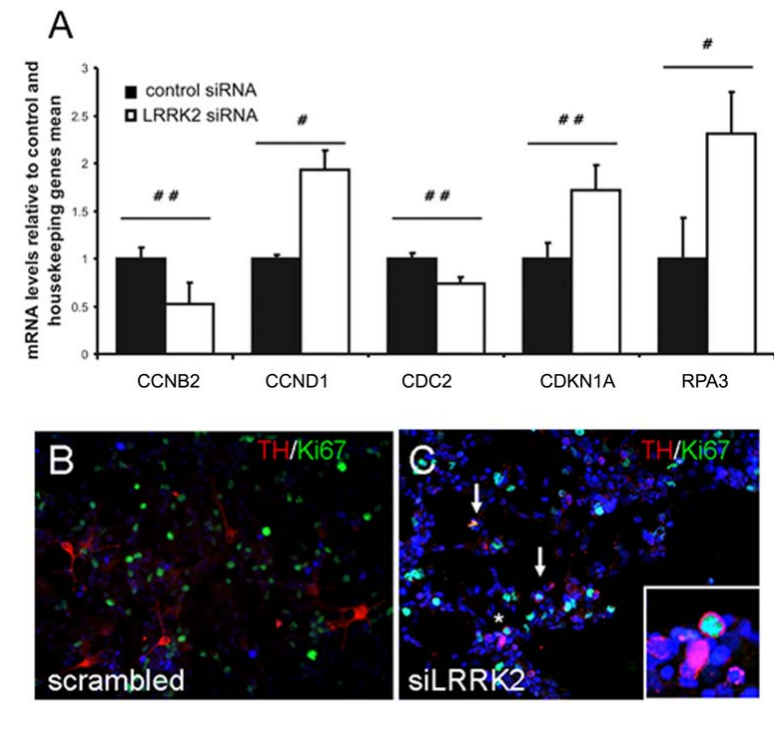
**LRRK2 deficient postmitotic DNs express proliferation marker**. LRRK2 siRNA-treated (white bars) or control (black bars) hmNPC RNA displayed activation or reduction of important cell-cycle regulators in differentiated cells (A). LRRK2 siRNA-treated or control (scrambled siRNA) hmNPCs were co-stained for TH and Ki67 (B, C). Note a reduction in LRRK2 expression and disappearance of protrusions that normally characterize healthy dopaminergic cells (C). Terminally differentiated TH^+ ^hmNPCs never exhibited Ki67 proliferation marker (B) which appeared in dopaminergic (TH^+^) siLRRK2 treated samples (arrows in C), particularly visible at higher magnification (insert corresponds to asterisk).

To further explore underlying reasons for cell death following LRRK2 protein down-regulation, signal transduction proteins from the MAPK, Akt/PKB and GSK signalling cascades were studied by antibody microarray analysis. In addition, cell cycle regulation, apoptosis and transcription factors were analyzed using the same method. Increased overall serine and tyrosine phosphorylation, increased concentrations of cyclin A, cyclin B1, cyclin D1, p18 and increased phosphorylation of apoptosis protein p53 (at Ser15) but non of CDKs was observed in hmNPCs expressing siLRRK2 versus scrambled siRNA (Figure 5). To ensure that the observed differences were due to the knockdown of LRRK2, we also calculated the variability between samples transfected with scrambled RNA which were less than 10 arbitrary units (a. u.) and were negligible (data not shown). The up-regulation of cyclin A, cyclin D1 and the early mitotic marker cyclin B1 suggests cell cycle reactivation provoked by LRRK2 down-regulation. Activation of p53 can lead to either cell cycle arrest and DNA repair or apoptosis [15]. Phosphorylation of p53 at Ser15 is usually induced by DNA damage and leads to a reduced interaction between p53 and its negative regulator, the oncoprotein MDM2 [16]. MDM2 inhibits p53 accumulation by targeting it for ubiquitination and proteasomal degradation [17]. Phosphorylation thus impairs the ability of MDM2 to bind p53, promoting both the accumulation and activation of p53 [18]. A PCR array analysis of 96 cell-cycle regulators revealed indeed increased (cyclin D1 [CCND1], cyclin-dependent kinase inhibitor 1A [CDKN1A] and replication protein A3 [RPA3]) or reduced (cyclin B2 [CCNB2] and cyclin-dependant kinase 1 [CDK1/CDC 2]) expression of five fundamental cell-cycle regulators (Fig. 6A). Cyclin D1 promotes cell-cycle progression and its overexpression is known to correlate with the early onset of cancer and risk of tumor progression and metastasis [19]. RPA3 is important for DNA synthesis and repair [20]. CDKN1A (p21/CIF1) is activated upon DNA-damage by the p53 pathway [21] strengthening the antibody microarray results. The complex of cyclin B/cdc-2 was originally defined as M phase-promoting factor, capable of inducing M phase in immature G2 oocytes [22], but cyclin B mRNA is also known to become instable after DNA damage [23]. In the same cultures, we recognized the proliferation marker Ki67 co-expressed in dying dopaminergic neurons (Fig. 6C). In parallel control cultures (scrambled siRNA), Ki67 was absent in TH-immunoreactive cells but present in other, presumably glial, cells (Fig. 6B).

## Discussion

In the present study we investigated a possible role of non-mutated LRRK2 for the differentiation and/or survival of human dopaminergic neurons. Widespread expression of LRRK2 protein throughout the normal brain suggests an important physiological role that has not been identified to date [24]. Consistent with the quite strong LRRK2 expression in the murine subventricular zone [25], a region of *in vivo *neurogenesis in the adult brain, we found high nestin/LRRK2 co-expression in expanded human mesencephalic neural progenitor cells (hmNPCs) *in vitro*. This characteristic of hmNPCs allowed us to knockdown the LRRK2 protein expression in progenitor cells using silencing RNA technology and to track their course of differentiation afterwards. siLRRK2 nucleofected hmNPCs produced less dopaminergic neurons when compared to scrambled controls possibly as a consequence of cell death driven by activated caspase-3 within DNs. Based on results obtained with comprehensive PCR array and antibody microarray analysis, we further postulate that the observed cell death results from cell cycle preservation or even reactivation in DNs. Most of the 44 examined proteins that were up-regulated in LRRK2 deficient differentiated hmNPCs belong to cell cycle-related proteins. Increased overall serine and tyrosine phosphorylation was also detected. Cell-cycle PCR array analysis likewise revealed regulation of five fundamental cell-cycle regulators. Together with the activation of other cell-cycle regulators, the down-regulation of cyclin B/cdc-2 by the LRRK2 knockdown suggests an activation of the cell cycle without the ability to progress through M-phase, an activation that might report DNA damage and might lead to apoptosis. Gene array experiments in neuroblastoma cells also revealed that knock-down of LRRK2 affects, among others, cell cycle genes and p53 signalling cascades [26]. Components of Wnt/β-catenin signalling pathway were also dysregulated along with 14 genes belonging to "nervous system development" category, highlighting the importance of LRRK2 for the whole brain and dopaminergic system development. Upregulation of p53 protein, plays a role in the degeneration of the nigro-striatal dopaminergic neurons in the parkinsonian brain [27]. Phosphorylation of p53, also detected in LRRK2 deficient hmNPCs, might influence cell death in dopaminergic neurons [28].

Healthy, terminally differentiated neurons do not proliferate, yet the expression of cell cycle markers, cyclins or cyclin dependent kinases (CDKs) in the postnatal or adult brain is still a matter of controversial debate [29,30]. Terminal differentiation is considered to be an irreversible state and neurons do not spontaneously re-enter the cell cycle, but they can be forced to do so. Studies in cell-culture systems and in *post-mortem *tissue from diseased human brain suggested a link between cell-cycle reactivation and neurodegeneration [31,32]. Pathologic DNs aberrantly expressed proliferating cell nuclear antigen (PCNA) and the transcription factor E2F-1, both *in vitro *and *in vivo*, following 1-methyl-4-phenylpyridinium (MPP^+^) intoxication, as well as in PD patients [33]. There is thus direct evidence of DNA replication preceding cell death in neurodegenerative diseases [34]. Another study on mutant tau-induced neurodegeneration in *Drosophila *indicated a role for cell-cycle activation downstream of tau phosphorylation subsequently leading to apoptosis [35]. Here, we demonstrate both, a total serine and tyrosine phosphorylation and cell death in DNs developed from LRRK2 deficient neuroprogenitors *in vitro*. In parallel, we recognized the proliferation marker Ki67 co-expressed in dying DNs that was not detected in control cells. *Substantia nigra *of PD patients showed a reduction of the number of LRRK2-immunoreactive cells when compared to healthy midbrain samples. The same is true for melanin containing cells, most of which are dopaminergic neurons. Both, LRRK2+ and LRRK2/melanin double-positive neurons are similar and significantly less in SN of PD patients in comparison to healthy SN, probably due to a common reduction of DA neurons in PD. This finding supports our hypothesis of LRKK2 expression in DA neurons. We did not perform double labelling in *post-mortem *human tissue (e.g. for TH to identify dopaminergic neurons) due to technical issues. Instead, we used melanin as a marker for dopaminergic neurons, although being aware that some phagocytosing cells may also contain melanin, which account for the modest reduction of melanin-containing cells in PD patients. On the other hand, the prominent reduction of LRRK2-immunoreactive cells, which was observed previously [9], compared to melanin containing cells may also indicate that down-regulation of LRKK2 precedes DNs death. This hypothesis is in line with our *in vitro *data showing siRNA knockdown of LRRK2 in human midbrain neural progenitors prior to differentiation, which also induced loss of DNs, most likely via activation of cell cycle genes and subsequent apoptosis. The generation of dopaminergic neurons in the adult brain is still a matter of discussion [36-38], which at present does not convincingly support adult dopaminergic neurogenesis [39]. Thus, if our findings of cell cycle activation and loss of dopaminergic neurons were of relevance to Parkinson's disease, a concept of cell cycle reactivation impairing survival mechanisms of dopaminergic neurons would be appropriate. However, our current experiments show merely that reducing the expression of LRRK2 in developing dopaminergic neurons reduces their number and induces apoptosis in remaining dopaminergic neurons associated with an activation of cell cycle genes.

It is still disputed whether mutations in the LRRK2 gene that account for familial PD reduce or increase the functional (especially kinase) activity of this protein. Some studies suggest that mutant LRRK2 induces a gain-of-function [6,40]. On the other hand, mutant LRRK2 also failed to promote protective effects conferred by the wild-type protein [41]. The latter findings are in agreement with our present data indicating that a lack of LRRK2 is critical during differentiation towards dopaminergic neurons. However, both findings do not preclude that there is a gain of kinase activity induced by the mutations.

Taken together, we hypothesize that insufficient LRRK2 might harm the differentiation and/or survival of dopaminergic neurons. LRRK2 seems to be involved in cell cycle regulation. Whether it directly inhibits cells cycle genes or acts indirectly via activators or suppressors of the cell cycle can not be answered based on our data. Future studies shall reveal which part of the complex cell cycle machinery is altered via LRRK2.

## Materials and methods

### Isolation, characterization and propagation of human NPCs (hNPCs)

Human fetal midbrain tissue was used to generate human midbrain NPCs cultures (hmNPCs). Samples were harvested and supplied by Advanced Bioscience Resources Inc., Alameda, CA, according to NIH and local IRB guidelines. Prior to experimental procedures primary cells from the ventral mesencephalon were characterized by FACS, immunocytochemistry and RT-PCR for markers of early and late DA differentiation as described. Cells were expanded for prolonged periods (> 10 passages) in reduced atmospheric oxygen (3%) [42,43].

### Differentiation of hmNPCs

Differentiation of hmNPCs was induced via replacement of expansion media by defined media without mitogens but with 2% B-27 (Invitrogen), 5 μM forskoline (Sigma-Aldrich Chemie GmbH Munich, Germany) and 100 pg/ml IL-1β (Sigma). Prior to immunostaining or protein extraction, hmNPCs were allowed to differentiate for 2 weeks.

### RNA interference treatment

We tested several different double-stranded siRNAs aimed to reduce expression levels of LRRK2 protein. The best effects were obtained with a target-specific LRRK2 siRNA and control siRNA-A (scrambled sequence siRNA) that were purchased from Santa Cruz (Santa Cruz Biotechnology, Inc., Santa Cruz, CA, USA). Both siRNAs were reconstituted under RNase-free conditions using the supplied buffers according to the protocol of the manufacturer. Delivery of siRNA into NPCs was performed with the Nucleofector technology using the mouse Nucleofector kit (Amaxa Biosystems, Koeln, Germany). Transfection efficiency was checked via transfecting the cells with Cy3-conjugated negative control siRNA duplex (QIAGEN, Hilden, Germany). hmNPCs were nucleofected either with LRRK2 siRNA or scrambled siRNA at the concentration of 100 pmol siRNA per 1 × 10^6 ^cells following by further 72 h expansion [44]. Alternatively, nucleofected cells were the next day initiated to differentiate for 2 weeks.

### Semiquantitative real-time RT-PCR analysis

Total cellular RNA was extracted from siRNA nucleofected NPCs using RNAeasy total RNA purification kit followed by treatment with RNase-free DNase (Qiagen, Hilden, Germany). Semi-quantitative real-time one step RT-PCR was carried out using the Stratagene system (MX3000P™ Stratagene, Heidelberg, Germany), and amplification was monitored and analyzed by measuring the binding of fluorescent SYBR Green I to double-stranded DNA. 1 μl (50 ng) of total RNA was reverse-transcribed and subsequently amplified using QuantiTect SYBR Green RT-PCR Master mix (Qiagen) and 0.5 μmol/l of both sense and antisense primers. The sequences for forward and reverse primers used for the target gene (TG) human *LRRK2 *were as follows: 5'-CTT GGC TTG GTC CTT TAT TTCC-3' and 5'-CCT GAG GCT GTT CCT TCT TCC-3'. The primers for the reference gene (RG) *HMBS *(hydroxymethylbilane synthase) were as follows: 5'-TCG GGG AAA CCT CAA CAC C-3' and 5'-CCT GGC CCA CAG CAT ACA T-3'. The efficiency of product formation by PCR was estimated from plots of Ct values versus serial dilutions, measured three times with different RNA samples. The relative RNA content was determined using the formula of the comparative cycle threshold (Ct): TG/RG = 2^Ct(RG)-Ct(TG) ^[45].

### PCR Superarray

Total RNA was extracted from siRNA nucleofected differentiated hmNPCs using RNAeasy total RNA purification kit followed by treatment with RNase-free DNase (Qiagen, Hilden, Germany). The pathway-focused (cell-cycle) RT2 Profiler™ PCR Array System (SABiosciences, Frederick, USA) was employed according to the manufacturer's manual. In short, cDNA was prepared, mixed with RT^2 ^qPCR Master Mix and aliquoted across the 96-well PCR array. Thermal cycling was done and collected data were subsequently analyzed using the provided analysis spreadsheet.

### Immunofluorescence

hmNPCs were grown on sterile glass cover slips, fixed with 4% paraformaldehyde in PBS for 10 min at room temperature and washed with PBS, counterstained with the DNA-binding dye 4'-6-Diamidino-2-phenylindole (DAPI, 2 μg/ml in PBS) followed by incubation in blocking buffer. The following primary antibodies were used: mouse monoclonal anti-nestin (Pharmingen, San Diego, CA, USA), rabbit polyclonal anti-β-tubulin III (anti-Tuj1; Covance, Freiburg, Germany), rabbit polyclonal anti-tyrosine hydroxylase (Santa Cruz Biotechnology, Inc., Santa Cruz, CA, USA), sheep polyclonal anti-tyrosine hydroxylase (Pel-Freez, Rogers, AK), rabbit polyclonal anti-Ki67 antigen (Novocastra Laboratories Ltd, Newcastle upon Tyne, UK); rabbit polyclonal anti-LRRK2 (NB300-267; Novus Biologicals, Littleton, CO, USA); rabbit polyclonal cleaved caspase-3 (Cell Signalling Technology, Inc., Danvers, USA). Finally, fluorescent secondary antibodies, Alexa Fluor^® ^488 conjugate or Alexa Fluor^® ^594 conjugate (Molecular Probes, Eugene, USA) were used. Coverslips were mounted onto glass slides and examined by a confocal laser scanning microscope (LSM 510, Zeiss, Oberkochen, Germany) at an excitation wavelength of 594 nm (helium/neon, red Alexa 594-immunofluorescence), and 488 nm (argon, yellow-green Alexa 488-immunofluorescence). Alternatively, glass slides were examined under a fluorescence microscope (Zeiss Axiovert 200). Acquisition of the immunostained cells was performed using the Image-analysis software AxioVision 4 (Carl Zeiss AG, Jena, Germany).

### Immunohistotochemistry

Two paraffin sections (5 – 6 μM; n = 2) of the midbrain were made from 4 patients with neuropathologic diagnosis of idiopathic PD and 4 controls, (mean age 64 ± 22 years), respectively. Sections were deparaffinized in Xylol for 3 × 10 min and hydrated in descending alcohol concentrations. After a microwave treatment with 10% citrate buffer (DAKO, Code # S2031) at 600 W 4 × 4 min and consecutive washing in PBS, sections were blocked in dry milk (5 g/l). Peroxidase blocking preceded overnight incubation in LRRK2 primary antibody (1:200, NB300-267; Novus Biologicals, Littleton, CO, USA). Incubation in biotinylated secondary antibody Link DAKO LSAB 2 System (k0675, Dako, Hamburg, Germany) followed by additional washing steps. Streptavidine-HRP DAKO LSAB 2 system was used for the Streptavidine conjugation. DAKO DAB (k3468, Dako, Hamburg, Germany) was used as substrate chromogen. Counterstaining was performed using Haemalaun (Mayer). Sections were dehydrated in ascending alcohol concentrations and Xylol and mounted in Entellan medium.

### Western blot analysis

Approximately 5 × 10^6 ^hmNPCs were harvested and lysed in 0.5 ml lysis buffer containing 10 mM HEPES-KOH (pH 7.9), 10 mM KCl, 1.5 mM MgCl_2_, 0.1% NP-40 and protease inhibitor cocktail (Roche). Running and blotting of proteins, membrane blocking and incubation with antibodies, as well as data analysis were performed essentially as reported [46]. Primary antibodies used for immunoblotting were as follows: mouse monoclonal anti-Nestin (Pharmingen); mouse monoclonal anti-GFAP (Chemicon International, Hampshire, UK); rabbit polyclonal anti-β-tubulin III (Covance); rabbit polyclonal anti-TH (Santa Cruz); rabbit polyclonal anti-LRRK2 (Novus); mouse monoclonal anti-actin (MP Biomedicals, Eschwege, Germany).

### Antibody microarray

The microarray analysis was performed using antibody microarrays *Signal.screen *44 F from the BIOSCORA GmbH (Leipzig, Germany). The microarray contains antibodies for detection of signal transduction proteins from the following signalling cascades: MAPK, Akt/PKB, GSK, cell cycle regulation, apoptosis and transcription factors (14 subarrays, 44 antibodies each, as well as 3 calibrator proteins/slide, totally 756 detection points/slide). Debris-free cell lysates (scrambled samples and siLRRK2 samples) were applied separately onto identical antibody subarrays printed on modified surface of the standard microscope glass slide. Protein concentration was 0,9 mg/ml for each sample. Protein binding and detection were performed according to experimental protocol of the manufacture. Read-out of signal intensities of the bound proteins (spot intensities) was performed using the Cy-3 (green) channel of the VersArray Microarray Scanner (Bio Rad). Signal intensities of the separate subarrays were measured using *Quanti.screen *microarray analysis software (BIOSCORA GmbH, Leipzig).

### Statistical analysis

Normally distributed data were subjected to statistical analyses as appropriate (t-test or ANOVA) using the SigmaStat software package (Jandel Corp., San Rafael, CA). Results are expressed as the mean ± S.E.M. Statistical significance was accepted at *P *< 0.05.

## Abbreviations

ANOVA: analysis of variance; a.u: arbitrary units; CDKs: cyclin-dependent kinases; DAPI: 6'-diamidino-2-phenylindole; DNs: dopaminergic neurons; EGF: epidermal growth factor; FGF-2: fibroblast growth factor 2; GFAP: glial fibrillary acidic protein; HMBS: hydroxymethylbilane synthase; hmNPCs: human mesencephalic neural progenitor cells; LRRK2: leucine-rich repeat kinase 2; MAPK: mitogen-activated protein kinase; MAPKKK: mitogen-activated protein kinase kinase kinase; MPP^+^: 1-methyl-4-phenylpyridinium; PD: Parkinson's disease; siRNA: small interfering ribonucleic acid; TH: tyrosine hydroxylase; Tuj1: Neuronal class III β-tubulin.

## Competing interests

The authors declare that they have no competing interests.

## Authors' contributions

JM did conception and design, carried out cell culture, siRNA studies, immunoblotting, immunocytochemistry, confocal microscopy, data analysis and interpretation, statistical analysis, performed manuscript writing and brought partial financial support. SCS contributed with provision of study material (NPC preparation and characterization) and carried out immunohistochemistry. VO provided samples of post mortem PD tissue and carried out immunohistochemistry. AKM performed PCR superarray analysis. AS participated in design and coordination of the study. JS conceived of the study and participated in its design and coordination. All authors read and approved the final manuscript.
